# Complex Composite Odontoma with Characteristic Histology

**DOI:** 10.1155/2013/157614

**Published:** 2013-06-15

**Authors:** Sujatha Govindrajan, J. Muruganandhan, Shaik Shamsudeen, Nalin Kumar, M. Ramasamy, Srinivasa Prasad

**Affiliations:** ^1^Department of Oral and Maxillofacial Pathology, Sri Venkateswara Dental College and Hospital, Thalambur, Chennai 603103, India; ^2^Department of Orthodontics, Sri Venkateswara Dental College and Hospital, Thalambur, Chennai 603103, India; ^3^Department of Oral and Maxillofacial Surgery, Sri Venkateswara Dental College and Hospital, Thalambur, Chennai 603103, India

## Abstract

Odontomas are the most commonly occurring odontogenic tumors, which are considered by many to be hamartomas rather than neoplasms. These clinically asymptomatic tumors are classified into complex and compound odontomas. They are usually discovered in radiographs and rarely cause bony expansion or infection. This paper discusses a case report of a complex odontoma exhibiting all the structural features and defects of enamel, dentine, and cementum in succession, with an overview on its etiology.

## 1. Introduction

Hamartomas of tooth forming tissues are termed as odontoma. They are the most common tumor of epithelial and mesenchymal origin and account for 22% of all odontogenic tumors [1]. This nonaggressive benign tumor contains enamel, dentin, cementum, and pulp either arranged in an orderly manner resembling a rudimentary tooth called compound odontoma or arranged in a haphazard manner called complex odontoma. Complex odontoma is less common when compared to the compound, and they present in ratio of 1 : 2 [2]. Odontomas rarely erupt in the oral cavity. We present a case of a partially erupted odontoma in the right third molar region.

## 2. Case Report

**Figure 1:**
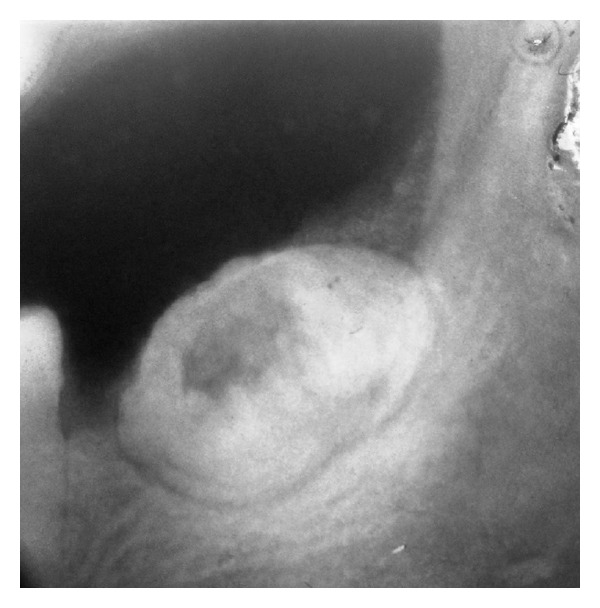
Radiographic image of the odontoma.

A 28-year-old male patient reported to a private clinic with a complaint of pain in the right lower posterior tooth region for about one week. On intraoral examination, a partially erupted tooth-like structure was seen. The pain was intermittent and was aggravated on chewing. Radiographic investigation revealed the presence of near-spherical opaque mass resembling calcified tissue measuring about 2 cm in diameter. The radiopaque mass with a density greater than bone and equal or greater than that of tooth was surrounded by a radiolucent rim in all areas except the erupted portion distal to normally erupted second molar (Figure 1).

**Figure 2:**
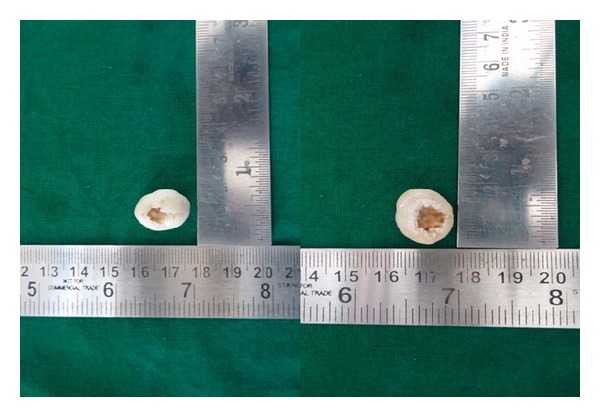
Superior and inferior surface of odontoma.

Removal of the mass was planned under local anaesthesia. Mucoperiosteal flap was raised distal to 47, and the calcified mass was removed. The spherical mass was about 2 cm in diameter with small irregular areas of indentations. The inferior side of the mass showed a hollow invagination giving the appearance of a small cup (Figure 2). The specimen was sent for histopathological examination. A diagnosis of complex odontoma was made clinically. 

**Figure 3:**
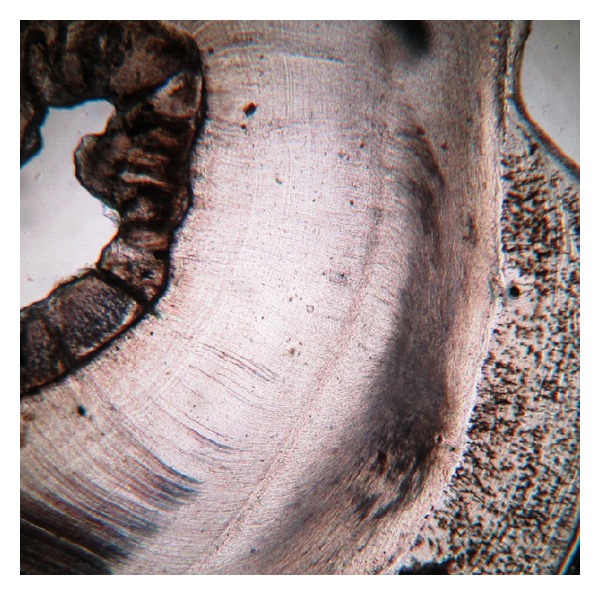
Enamel, dentine and cementum in succession (photomicrograph, ground section 10x).

**Figure 4:**
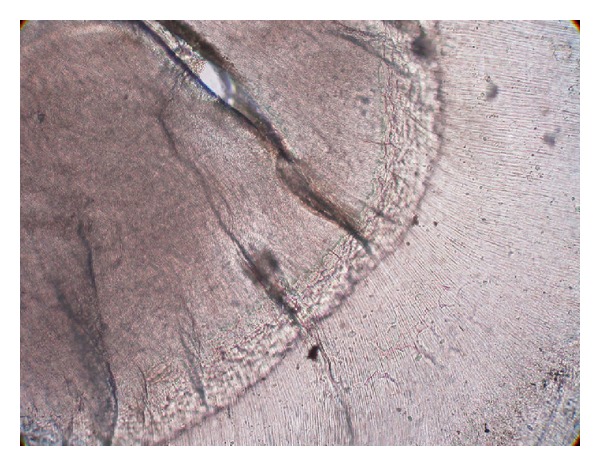
Presence of lamellae, spindles and tufts (photomicrograph, ground section 10x).

**Figure 5:**
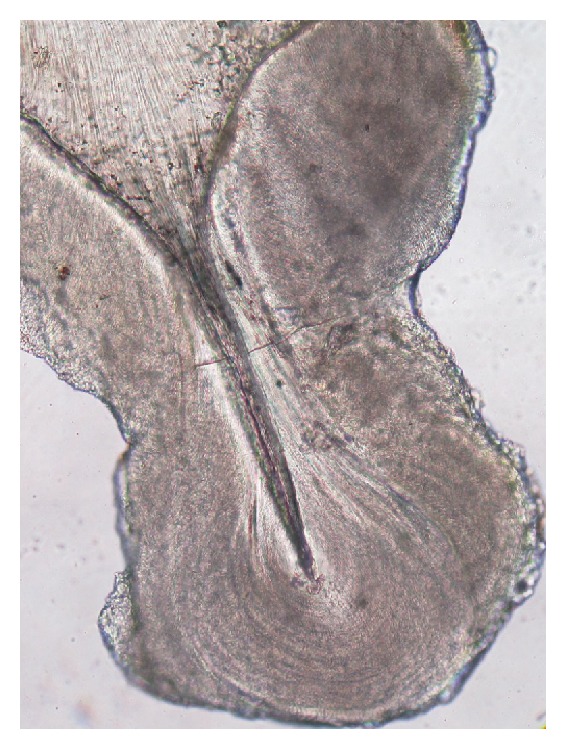
Incremental lines of enamel (photomicrograph, ground section 10x).

**Figure 6:**
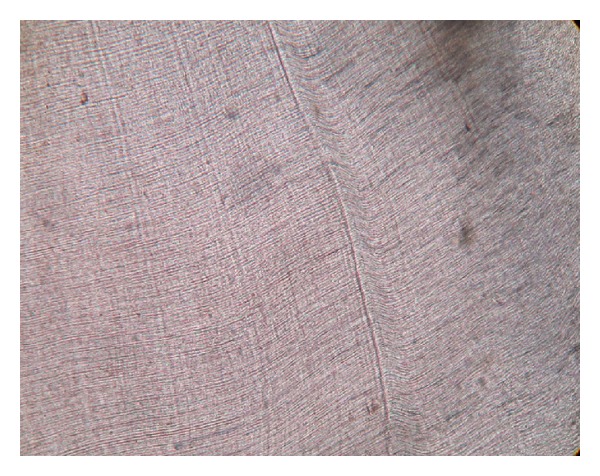
Dentinal tubules with incremental lines (photomicrograph, ground section 10x).

Ground sections were done on the dissected halves of the hard tissue mass. The ground section showed areas of enamel, dentine, and cementum in succession (Figure 3). The enamel showed uneven thickness and undulating surface. Hypocalcified areas like lamellae, spindles, tufts, and incremental lines were seen (Figures 4 and 5). Some areas showed irregular rod patterns and gnarled enamel (Figure 6).

**Figure 7:**
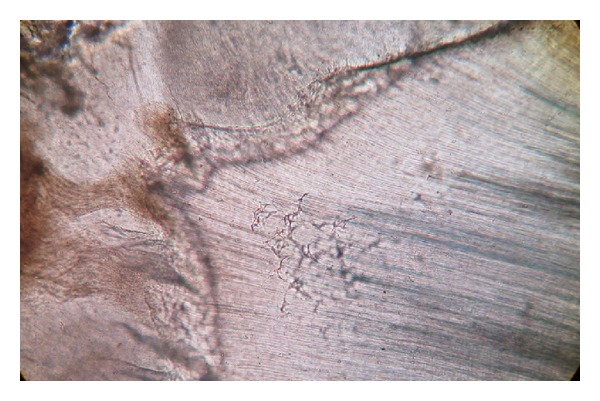
Interglobular dentine (photomicrograph, ground section 10x).

**Figure 8:**
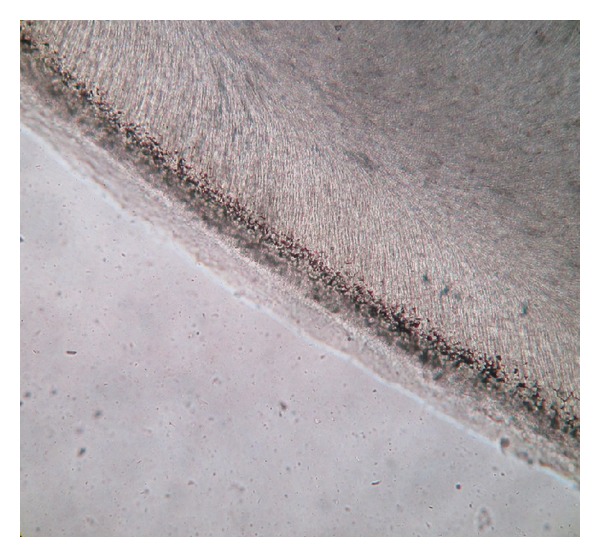
Tomes granular layer (photomicrograph, ground section 10x).

**Figure 9:**
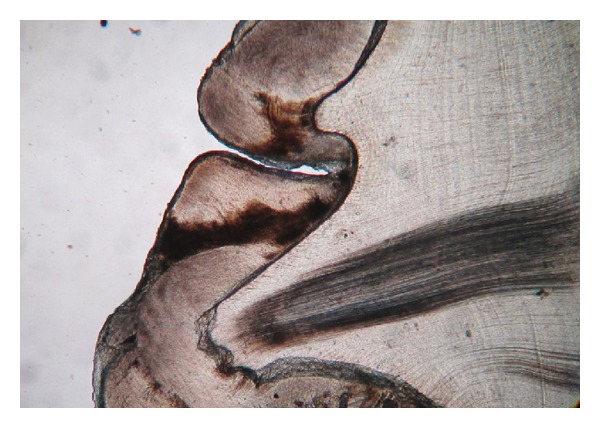
Dead tracts and gnarled enamel (photomicrograph, ground section 10x).

**Figure 10:**
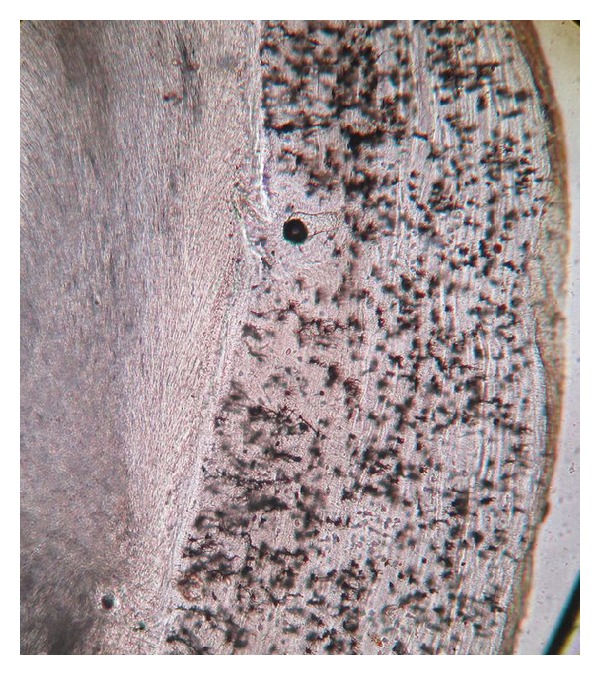
Cemetocytes with canaliculi (photomicrograph, ground section 10x).

The dentinoenamel junction was regular and scalloped in some areas. The underlying dentine showed “S” shaped dentinal tubules. Primary and secondary dentine types were observed with clearly visible incremental lines (Figure 7). Hypocalcified areas like interglobular dentine and Tomes' granular layer were also observed (Figures 8 and 9). Dead tracts were also seen (Figure 9). Next to the dentine there was a layer of cementum of varying thickness. Both types of cementum were observed. The cellular cementum was more prominent with numerous cementocytes. Each cementocyte had about 8–10 canaliculi, which are branching and oriented away from the dentinal surface (Figure 10).

## 3. Discussion

Paul Braco in 1867 was the first to coin the term odontoma and defined the term as tumors formed by the overgrowth or transition of complete dental tissue [3]. It is a growth in which both the epithelial and mesenchymal cells exhibit complete differentiation resulting in formation of enamel and dentin by the functional ameloblasts and odontoblasts. These odontogenic cells are usually disorganized, and the enamel, dentine, and pulpal tissue are laid down in an abnormal pattern [4]. Our case represented the presence of functional ameloblast, odontoblast, and cementoblast by formation of these normal hard tissue structures. WHO has classified four tumors which arise from mixed tissue origin giving normal enamel and dentine formation, odontoma being the most common of them [5].

According to WHO, this lesion is “a malformation in which all the dental tissues are represented, individual tissues being mainly formed but occurring in more or less disorderly pattern.” When the calcified tissues are simply arranged in an irregular mass bearing no morphological similarity to rudimentary teeth, they are termed complex odontoma. A compound odontoma comprises calcified structures arranged in an orderly pattern that result in many teeth-like structures, but without morphological resemblance to normal tooth [6]. Our case presented as an irregular mass and was classified as a complex odontoma.

The etiology of odontoma is not clear, and various causes like local trauma, infection, hereditary anomalies like Gardner's syndrome, Hermann's syndrome, odontoblastic hyperactivity, and alteration in genetic components responsible for tooth development are also considered [7]. Hitchin suggested that odontomas are inherited (through a mutant gene) due to interference, possibly postnatal, with genetic control of tooth development [8]. Experimental studies in rats suggested the role of trauma in formation of odontomas [9].

Sources of cells for odontomas could be mature ameloblasts, cell rests of Serres, or extraneous odontogenic epithelial cells [1, 10–12]. These cells can be stimulated by either environmental or genetic factors. Genetic factors could be either due to inheritance of abnormal genes or mutation of the responsible genes and interference in the mechanism of genes controlling tooth formation [7].

 Environmental factors like trauma, growth pressure, and infection may play a role in the pathogenesis of odontoma. A vertically directed force in the form of trauma which is directed to the permanent tooth bud through the deciduous tooth can lead to morphological changes in the permanent tooth bud leading to formation of odontomas [13]. Studies by Glasstone (1952) and Rushton (1957) have supported the role of trauma in the development of odontoma [8]. Levy states that the stage of development during which the trauma has occurred determines the development of hypoplastic teeth, odontomas, and supernumerary tooth [9]. Presence of odontoma in sites other than tooth bearing regions suggests that trauma could have possibly displaced the developing tooth germ leading to its malformation [14].

Growth pressure due to inadequate space is been quoted as the etiology in some odontomas. This theory by Hitchin states that abnormal pressure from the deciduous roots over the developing tooth germ could lead to the formation of odontomas [8, 15, 16]. Infections can cause the division of tooth germ or interfere with tooth development [7].

Odontomas were discovered at any age but the most prevalent age of detection was the second decade of life with a slight predilection for occurrence in males when compared to females [17]. Compound odontomas are more common in the anterior segment of the jaws (61%), and complex odontomas are more common in the posterior segment (59%), with higher occurrence in the right than in the left side [4]. Compound odontomas are commonly seen in the incisor-cuspid region of maxilla and complex type in the premolar-molar region of mandible [18]. In our case, the patient was a middle aged male patient with the complex odontoma occurring in the right posterior segment of the mandible.

Odontomas are clinically asymptomatic, although occasionally retention of deciduous teeth, noneruption, swelling, pain, tooth displacement, cortical bone expansion, and infection may be noted [19]. Alterations to neighboring teeth such as malpositioning, devitalisation, malformation, aplasia, and retained teeth were observed in 70% of the cases [20]. Odontomas are classified as intraosseous when seen totally embedded in the bone with or without signs of eruption and extraosseous when present in the soft tissues over the tooth bearing bone [21]. 

Eruption of odontomas is different from a normal tooth as there is lack of periodontal ligament. As there is no root formation, the increasing size of the odontoma leads to sequestration of the overlying bone by causing pressure and eventually occlusal movement and eruption [22]. Bone remodeling is considered as the other possible factor in initiating eruption. Cellular activity in reduced enamel epithelium and dental follicles is required for eruption. Epidermal growth factor β [EGF-β] and transforming growth factor [TGF] are expressed by signals from reduced enamel epithelium, helping in production of colony-stimulating factor by the follicular cells, recruiting osteoclasts to the follicle. Proteases secreted by reduced enamel epithelium also help in breakdown of follicle and produce a path of least resistance [23].

Diagnosis of odontomas is usually by radiographs. A routine intraoral radiograph helps in diagnosing the presence of odontoma and the classification is usually by visualization. A compound odontoma, in a radiograph shows well-organized malformed tooth or tooth-like structures in a radiolucent follicle-like space whereas the complex type shows irregularly shaped radiopacity surrounded by a radiolucent rim. In cases where there are numerous tooth-like structures in a compound odontoma, differentiating the two becomes difficult [7]. If both visual and radiographic images fail to give a definite diagnosis as in some cases, other procedures like histologic examination are used. In our case, though we established a diagnosis of complex odontoma through radiographic and visual examination, we decided to see the histological features through ground section.

Ground section usually reveals the presence of all hard tissues in succession representing enamel, dentine, and cementum. Hypocalcified areas of both enamel and dentine are seen. In our case, the microscopy showed remarkable features that make for interesting discussion. Treatment of odontomas is usually by conservative surgical removal and preservation of impacted or embedded tooth if present.

## 4. Conclusion

Odontomas are common odontogenic tumors, which are usually asymptomatic and are diagnosed by routine radiographs. Etiology of this tumor is not clear, and López-Areal in his study has supported the role of injury during childhood for formation of odontomas [24]. In our case, though the patient did not give a clear history of childhood injury, presence of the normal structures in an orderly pattern gives us an opinion that some mild interference during formation of third molar has occurred.
